# A comprehensive transcript index of the human genome generated using microarrays and computational approaches

**DOI:** 10.1186/gb-2004-5-10-r73

**Published:** 2004-09-23

**Authors:** Eric E Schadt, Stephen W Edwards, Debraj GuhaThakurta, Dan Holder, Lisa Ying, Vladimir Svetnik, Amy Leonardson, Kyle W Hart, Archie Russell, Guoya Li, Guy Cavet, John Castle, Paul McDonagh, Zhengyan Kan, Ronghua Chen, Andrew Kasarskis, Mihai Margarint, Ramon M Caceres, Jason M Johnson, Christopher D Armour, Philip W Garrett-Engele, Nicholas F Tsinoremas, Daniel D Shoemaker

**Affiliations:** 1 Rosetta Inpharmatics LLC, 12040 115th Avenue NE, Kirkland, WA 98034, USA; 2 Merck Research Laboratories, W42-213 Sumneytown Pike, POB 4, Westpoint, PA 19846, USA; 3 Rally Scientific, 41 Fayette Street, Suite 1, Watertown, MA 02472, USA; 4 Amgen Inc, 1201 Amgen Court W, Seattle, WA 98119, USA; 5 The Scripps Research Institute, Jupiter, FL 33458, USA

## Abstract

**Background:**

Computational and microarray-based experimental approaches were used to generate a comprehensive transcript index for the human genome. Oligonucleotide probes designed from approximately 50,000 known and predicted transcript sequences from the human genome were used to survey transcription from a diverse set of 60 tissues and cell lines using ink-jet microarrays. Further, expression activity over at least six conditions was more generally assessed using genomic tiling arrays consisting of probes tiled through a repeat-masked version of the genomic sequence making up chromosomes 20 and 22.

**Results:**

The combination of microarray data with extensive genome annotations resulted in a set of 28,456 experimentally supported transcripts. This set of high-confidence transcripts represents the first experimentally driven annotation of the human genome. In addition, the results from genomic tiling suggest that a large amount of transcription exists outside of annotated regions of the genome and serves as an example of how this activity could be measured on a genome-wide scale.

**Conclusions:**

These data represent one of the most comprehensive assessments of transcriptional activity in the human genome and provide an atlas of human gene expression over a unique set of gene predictions. Before the annotation of the human genome is considered complete, however, the previously unannotated transcriptional activity throughout the genome must be fully characterized.

## Abstract 2

A combination of microarray data with extensive genome annotations resulted in a set of 28,456 experimentally supported transcripts, providing the first experiment-driven annotation of the human genome.

## Background

The completion of the sequencing of the human, mouse and other genomes has enabled efforts to extensively annotate these genomes using a combination of computational and experimental approaches. Generating a comprehensive list of transcripts coupled with basic information on where the different transcripts are expressed is an important first step towards annotating a genome once it has been fully sequenced. The task of identifying the transcribed regions of a sequenced genome is complicated by the fact that transcripts are composed of multiple short exons that are distributed over much larger regions of genomic DNA. This challenge is underscored by the widely divergent predictions of the number of genes in the human genome. For example, direct clustering of human expressed sequence tag (EST) sequences has predicted as many as 120,000 genes [1], whereas sampling and sequence-similarity-based methods have predicted far lower numbers, ranging from 28,000 to 35,000 genes [2-5], and a hybrid approach has suggested an intermediate number [6]. Furthermore, the availability of a completed draft sequence of the human genome has yielded neither a proven method for gene identification nor a definitive count of human genes. Two initial analyses of the human genome sequence that used strikingly different methods both suggested the human genome contains 30,000 to 40,000 genes [2,3]. However, a direct comparison of the predicted genes revealed agreement in the identification of well-characterized genes but little overlap of the novel predictions. Specifically, 84% of the RefSeq transcripts agreed with fewer than 20% of the predicted transcripts matching between the two analyses. This result suggests that, individually, these datasets are incomplete and that the human genome potentially contains substantially more unidentified genes [7].

Several recent studies have highlighted the limitations of relying solely on computational approaches to identify genes in the draft of the human genome [8-13]. Furthermore, substantial experimental data from direct assays of gene expression provide evidence for many genes that would not have been recognized in the analyses just mentioned. Saha and colleagues used a new LongSAGE technology to provide strong evidence that there are thousands of genes left to be discovered in the human genome [9]. Specifically, they sequenced over 27,000 tags from a human colorectal cell line that collapsed down to 5,641 unique groups. Interestingly, only 61% (3,419) of the tags matched known or predicted genes, whereas 10% (575) matched novel internal exons and 14% (803) appear to represent completely novel genes [9]. They extrapolate from these data to predict as many as 7,500 exons from previously unrecognized genes. A recent analysis by Camargo *et al*. [8] also indicates that we are far from defining a complete catalog of human genes based on the analysis of 700,000 ORESTES (Open Reading Frame ESTs) that were recently released into GenBank. Finally, Kapranov and colleagues recently constructed genome-tiling arrays for human chromosomes 21 and 22 to comprehensively query transcription activity over 11 human tissues and cell lines [10]. They detected significant, widespread expression activity over a substantial proportion of these chromosomes outside of all known and predicted gene regions.

Most current methods in widespread use for identifying novel genes in genomic sequence depend on sequence similarity to expressed sequence and protein data. For example, *ab initio *prediction programs operate by recognizing coding potential in stretches of genomic sequence, where the recognition capability of these programs depends on a training set of known coding regions [14]. Therefore, genes identified by *ab initio *prediction programs or assembled from EST data are also inaccurate or incomplete much of the time [10-12]. While *ab initio *prediction programs perform well at identifying known genes, predictions that do not use existing expressed sequence and protein data often miss exons, incorrectly identify exon boundaries, and fail to accurately detect the 3' and 5' untranslated regions UTRs [14]. Similarly, EST data may be biased towards the 3' or 5' UTR [13]. These deficiencies are addressed in full-length gene cloning strategies [13], but cloning is still a laborious process which could be accelerated if we were able to start from a more accurate view of a putative gene [13].

Recently, several groups have used microarrays to test computational gene predictions experimentally and to tile across genomic sequence to discover the transcribed regions in the human and other genomes [10-12,15-17]. These array-based approaches detected widespread transcriptional activity outside of the annotated gene regions in the human, *Arabidopsis thaliana *and *Escherichia coli *genomes. The recent sequencing and analysis of the mouse genome indicates extensive homology between intergenic regions of the human and mouse genomes, further highlighting the potential for other classes of transcribed regions [18]. Interestingly, recent tiling data suggests that many of these conserved intergenic regions are transcribed [15,16].

In the study reported here, we describe hybridization results generated from two large microarray-based gene-expression experiments involving predicted transcript arrays spanning the entire human genome and a comprehensive set of genomic tiling arrays for human chromosomes 20 and 22. mRNA samples collected from a diversity of conditions were amplified using a strand-specific labeling protocol that was optimized to generate full-length copies of the transcripts. Analyses of the resulting hybridization data from both sets of arrays revealed widespread transcriptional activity in both known or high-confidence predicted genes, as well as regions outside current annotations. The results from this analysis are summarized with respect to published genes on chromosomes 20 and 22 in addition to our own extensive set of genome alignments and gene predictions. Combining computational and experimental approaches has allowed us to generate a comprehensive transcript index for the human genome, which has been a valuable resource for guiding our array design and full-length cloning efforts. In addition, the expression data from the 60 conditions provides a comprehensive atlas of human gene expression over a unique set of gene predictions [19].

## Results

### Generating a comprehensive transcript index of the human genome

**Figure 1:**
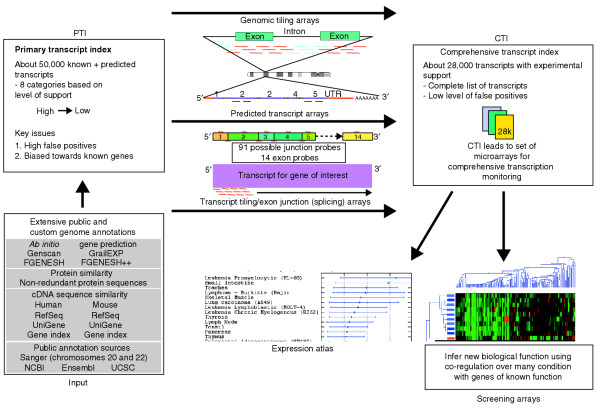
A process to generate a comprehensive transcript index (CTI) for the human genome. The first step is the assembly of a comprehensive set of annotations to generate a predicted transcript index (PTI). Sets of microarrays capable of monitoring the transcription activity over the entire genome can then be designed on the basis of the PTI. The different microarray types that can be used in this process include predicted transcript arrays (PTA), exon junction arrays (EJA) [21] and genome tiling arrays (GTA). After hybridizing a diversity of conditions onto these arrays, the transcription data are processed to identify a comprehensive set of transcripts (the CTI) and associated probes that are capable of querying all forms of transcripts that may exist in the genome. This set of probes comprises a focused set of microarrays that can be used in more standard microarray-based experiments.

Figure 1 illustrates the process we used to generate a comprehensive transcript index (CTI) for the human genome that represents just over 28,000 known and predicted transcripts with some level of experimental validation. The first step in this process was to generate a 'primary transcript index' (PTI) by mapping a comprehensive set of computationally and experimentally derived annotations onto the genomic sequence. The computational predictions include the output of gene-finding algorithms and protein similarities, while the experimentally derived alignments are based on ESTs, serial analysis of gene expression (SAGE), and full-length cDNAs. The resulting list of transcripts in the PTI can be loosely ranked or classified into different categories, ranging from high confidence to low confidence, on the basis of the level of underlying experimental support. The advantages of a PTI are that the computations can be performed on a genome-wide scale and it incorporates the massive amounts of publicly available EST, SAGE and cDNA sequence data. However, the resulting transcript index has two significant limitations. First, the *ab initio *gene-finding algorithms tend to have a high false-positive rate when applied at a low-stringency setting to cast as broad a discovery net as possible. Second, gene-finding algorithms are trained on known protein-coding genes, which may limit their ability to detect truly novel classes of transcribed sequences.

The second step towards the CTI is the use of two different types of microarrays to address these limitations (Figure 1). First, predicted transcript arrays (PTA) were used to determine experimentally which of the lower-confidence predictions in the PTI were likely to represent real transcripts. Second, genomic tiling arrays were used to survey transcriptional activity in a completely unbiased and comprehensive fashion. As shown in Figure 1, the CTI plays a central part in the subsequent design of screening arrays. These are used to monitor RNA levels for all the transcripts across a large number of diverse conditions to begin the process of assigning biological functions to novel genes based on co-regulation with known genes [20]. The CTI is also used to design exon/junction arrays that can be used to discover and monitor alternative splicing across different tissues and stages of development [21].

### Generating a PTI

To generate the PTI, three distinct computational analysis steps were executed in parallel: predictions based on similarity to expressed sequences from human and mouse; predictions based on similarity to all known proteins; and *ab initio *gene predictions. The process resulted in mapping 91% of the well characterized genes found in the RefSeq database [22], a percentage consistent with initial genome annotation results [2,3]. The mapping results were generated by collapsing overlapping gene models and regions of similarity to define locus projections, which comprise the distinct transcribed regions making up our PTI. While the reliance on gene predictions and protein alignments biases the PTI towards protein-coding genes, the alignment of all expressed sequences should represent many of the non-coding genes reported to date. A comprehensive index of non-coding genes would require tiling arrays, as described later.

All locus projections were classified into one of eight categories on the basis of the level of underlying evidence from expressed sequence similarity, protein similarity and *ab initio *predictions. The categories, in decreasing order of support, are as follows: (1) known genes, taken as the set of 11,214 human genes represented in the RefSeq database when the arrays were designed; (2) *ab initio *gene models with expressed sequence and protein support; (3) *ab initio *gene models with expressed sequence support; (4) *ab initio *gene models with protein support; (5) alignments of expressed sequence and protein data; (6) alignments of expressed sequence data, requiring at least two overlapping expressed sequences; (7) *ab initio *gene models with no expressed sequence or protein support; and (8) alignments of protein data. Because of the limitations discussed in the previous section, we considered predictions with a single line of evidence (categories 6-8) as low confidence.

Table 1 provides summaries resulting from a comparison between our PTI and the published Sanger Institute data for chromosomes 20 and 22 [23,24]. Our locus projections overlap 1,177 of 1,297 (91%) Sanger genes on chromosome 20 and 854 of 936 (91%) Sanger genes on chromosome 22, and our predicted exons overlap 7,306 of 7,556 (97%) and 4,819 of 5,014 (96%) total Sanger chromosome 20 and 22 exons, respectively. This comparison highlights the fact that our annotations result in the detection of both genes and exons in genomic sequence with high sensitivity.

### Predicted transcript arrays

We previously described a high-throughput, experimental procedure to validate predicted exons and assemble exons into genes by using co-regulated expression over a diversity of conditions [11]. Here we employ a similar strategy over the entire genome by hybridizing RNA from 60 diverse tissue and cell-line samples to a set of arrays designed from the PTI. For a complete list of the transcripts represented on the predicted transcript arrays and 60 tissues and cell lines hybridized to these arrays (see Additional data files 1 and 2). We designed two probes per exon, where possible, for exons containing the highest-scoring probes as described in the methods from each transcript in our PTI set (on average, a total of four probes per transcript). This was done to balance the poor specificity of *ab initio *gene-finding algorithms [14,25,26] against the significant microarray costs associated with large-scale gene-expression experiments. The resulting hybridization data provides experimental validation of those low-confidence predicted genes that are either unsupported or minimally supported by existing EST data, thereby providing a means of determining which transcripts are included in the CTI.

### Summary of predicted transcript validation on chromosomes 20 and 22

We used an enhanced version of a previously described gene-detection algorithm to analyze the predicted transcript array dataset [11]. Basically, the hybridization data from probes each transcript from the PTI were examined to identify those transcripts with probes that appear to be more highly correlated over the 60 diverse conditions. Transcripts with probes that behaved similarly over the different conditions tested were considered to be expression-validated genes (EVGs). Unlike our original algorithm that used Pearson correlations to group similarly behaving probes, our enhanced algorithm incorporated a probe-specific model to assess the most likely set of probes making up a transcriptional unit [27] (see Materials and methods for details). We used the extensive publicly available annotations on chromosomes 20 and 22 to assess the sensitivity and specificity of our array-based detection procedure.

The sensitivity of our procedure was assessed by computing the EVG detection rate for those Sanger genes that overlap predictions (locus projections) represented in our PTI (Table 2). The average detection rate for our locus projections on chromosomes 20 and 22 is approximately 70% for those overlapping Sanger genes and just over 80% for those locus projections derived from RefSeq alignments (locus category = known) that represent Sanger genes. A true positive in this instance was defined as an expression-verified gene containing at least two probes, where at least one of the probes was contained within the exon of a Sanger or RefSeq gene.

This 20% false-negative rate is the result of a complex mixture of issues, including limitations in our EVG-detection algorithm, limitations in the probe design step, lack of expression in the conditions profiled, and/or alternative splicing events. While the EVG-detection algorithm provides an efficient method to assemble probes into transcript units, the detection capabilities of this model could be expected to improve as the number of samples and the number of probes targeting any given transcript increases. The use of four probes per predicted transcript was determined to be sufficient for detection of most transcripts, as supported by the overall detection rate of known genes, although in many cases the probe design step was limited by our ability to find four high-quality probes per transcript. For many transcripts, there were not four nonoverlapping probes predicted to have good hybridization characteristics for the microarray experiment carried out here. The 60 samples were chosen to represent a broad array of tissue types, as an exhaustive list of human tissues is impossible to obtain. Because no replicate tissues/cell lines were run for any of the 60 chosen samples, we relied on the replication inherent in monitoring the same transcripts over 60 different conditions. In this case, genes expressed in multiple samples provide the replication necessary to increase our confidence in the detections. However, there are clear limitations in not replicating tissues/cell lines, as genes may be expressed in only a single condition or may be switched on only under certain physiological conditions or only during a certain stages of development. In such cases, we would have reduced power to detect these genes.

Genes in the lower-confidence categories of our PTI annotations, which are not typically considered genes by Sanger, were detected at a significantly reduced rate. Interestingly, of the 337 (188 +149) higher-confidence transcripts on chromosomes 20 and 22 that did not intersect with Sanger genes, 47 (or 14%) were detected as EVGs (Table 2). These transcripts represent potential novel transcripts on these two highly characterized chromosomes.

However, before we can make claims to the discovery potential for this method over the entire genome, we need to assess the false-positive detection rates. To this end, we defined as false positives all detections made in regions with support by only a single gene model that fell outside Sanger-annotated genes on chromosomes 20 and 22. Applying this definition over all transcripts in our PTI leads to a false-positive rate of 3% (11 out of 406). Because we cannot exclude the possibility that some of the transcripts supported by a single gene model represent real genes, we consider this false-detection rate as an upper bound on the actual false-positive rate. Accepting that the Sanger annotations represent the gold standard for chromosome 22, we detected 70% of all Sanger-annotated genes, while only 4% of the chromosome 22 locus projections that did not intersect Sanger genes were detected by our procedure, highlighting the sensitivity and specificity of this approach. In addition, the enrichment for EVG detections in Sanger genes versus the non-Sanger PTI on chromosomes 20 and 22 was extremely significant with a *p*-value effectively equal to 0 when using the chi-square test for independence (*χ*^2 ^= 3,093, with 1 degree of freedom (df)).

Summarizing EVG data over the entire genome and assessing the discovery potential. The last column of Table 2 provides the number of expression verified genes detected over the entire genome for locus projections in our PTI. This represents the most comprehensive direct experimental screening of *ab initio *gene predictions ever undertaken. We can use the false-positive and negative rates derived above to assess the discovery potential on that part of the genome that has not been as extensively characterized as chromosomes 20 and 22. First, we note that our detection rates over the genome were similar to that given for chromosomes 20 and 22. That is, 75% of the category 1 genes (RefSeq genes) were detected over the entire genome, compared to 80% for chromosomes 20 and 22. In total, 15,642 genes in the PTI were experimentally validated using this array-based approach. Assuming the false-positive rate of 3% defined above and a conservative false-negative rate of 30%, defined as the percentage of Sanger genes we failed to detect on chromosomes 20 and 22, these data suggest there are close to 21,675 potential coding genes represented in our PTI set. Because our PTI misses close to 10% of the Sanger genes, we corrected this number for those genes not represented in this set and provide an estimate of the total number of protein-coding genes in the human genome supported by our data to be approximately 25,000. This number is consistent with estimates given in the current release (22.34d.1) of the Ensembl database [28,29].

However, we caution that the estimate provided is based solely on the data described here, and that orthogonal sources of data [30] continue to suggest that the actual number of genes will be known only after the transcriptome has been completely characterized.

**Figure 2:**
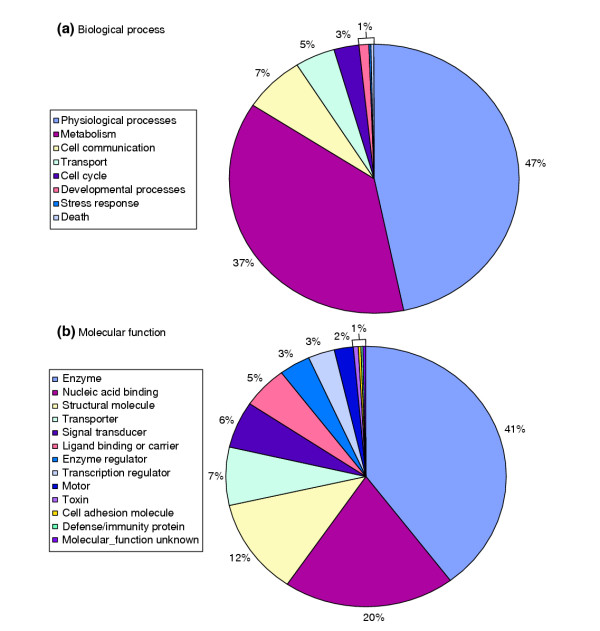
Gene Ontology (GO) classification of novel expression-validated genes (EVGs). EVGs not supported by the expressed sequence data (2,093) were submitted to a search against the Pfam database. Those with significant alignments (339) were assigned GO codes based on Pfam. The pie charts show the distribution of GO terms within this set of EVGs. Note that the total number of GO terms in each category is greater than the number of EVGs because of assignment of multiple GO terms to some EVGs. **(a) **Distribution of the different 'biological process' GO codes assigned to the EVGs with significant hits to the Pfam database: a total of 526 GO terms. **(b) **Distribution of the different 'molecular function' GO codes assigned to the EVGs with significant hits to the Pfam database: a total of 374 GO terms.

From Table 2 we note that 2,093 (1,428 + 555 + 110) of the transcripts that were detected as EVGs had only one line of evidence (EST alignment, protein alignment or *ab initio *prediction). These 2,093 transcripts represent a rich source of potential discoveries in our PTI. To assess the potential biological functions of this novel gene set, we annotated translations of this set by searching the domains represented in the Protein Families database (Pfam) [31]. The search results were used to assign each of the translations to Gene Ontology (GO) [32] codes as described in the methods. Figure 2 graphically depicts the breakdown of the most common GO codes for two of the three major GO categories. These data suggest there may still be a significant number of protein-coding genes with important biological functions, given that domains/motifs represented in these predicted genes are similar to those found in known genes. The 339 predictions that were validated as EVGs and that had protein domains of biological interest would be natural candidates for full-length cloning, over the 24,532 (7,170 + 16,822 + 540 from Table 2) other lower-confidence predictions in our set.

### EVG data as an expression index

**Figure 3:**
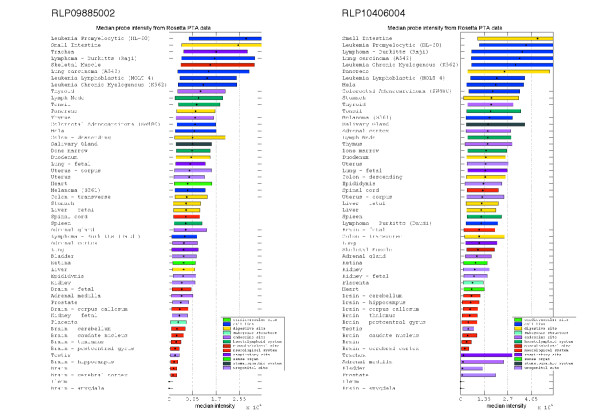
Utilizing PTA data as an expression index. Absolute transcript abundance over the 60 conditions described in [19] for two expression-supported transcripts. RLP09885002 represents a known gene (*ATP1A1*, ATPase, Na^+^/K^+ ^transporting, alpha 1 polypeptide) whereas RLP10406004 was supported solely by gene model predictions before microarray validation.

Because multiple probes in each of the approximate 50,000 predicted genes in the human genome have been monitored over 60 different tissues and cell lines, the EVG data represent a significant atlas of human gene expression that is now publicly available [19]. For each transcript, the intensity information from the corresponding probes was optimally combined as described by Johnson *et al. *[21] to provide a quantitative measure of the relative abundance across the panel of 60 conditions, as shown in Figure 3.

### Tiling arrays for chromosomes 20 and 22

To complement the use of PTI arrays, we constructed a set of genome tiling arrays comprised of 60 mer oligonucleotide probes tiled in 30 base-pair steps through both strands of human chromosomes 20 and 22. Repetitive sequences identified by RepeatMasker were ignored for probe design. These genome tiling arrays allow for an unbiased view of the transcriptional activity outside of known and predicted genes on these two chromosomes. mRNA from six (chromosome 20) or eight (chromosome 22) conditions was amplified and hybridized to the tiling arrays (see [19] and Additional data files 3 and 4). As with the PTI arrays, the amplification protocol generated strand-specific cDNA copies of the transcripts, which were full-length. Using a two-step procedure, the resulting data were analyzed to detect sequences expressed in at least one condition [33]. First, we examined probe behavior over conditions in overlapping windows of size 15,000 bp to identify windows that probably contained transcribed sequences, using a robust principal component analysis (PCA) method [33]. Second, for regions identified as likely to contain transcribed sequences, we attempted to discriminate between probes corresponding to expressed sequences (expressed 'exons') and probes corresponding to untranscribed sequences ('introns' or intergenic sequence) using a clustering procedure on variables derived from the PCA procedure [33]. All analysis results derived from this procedure were interpreted in the light of the Sanger annotations and our custom PTI set described above.

**Figure 4:**
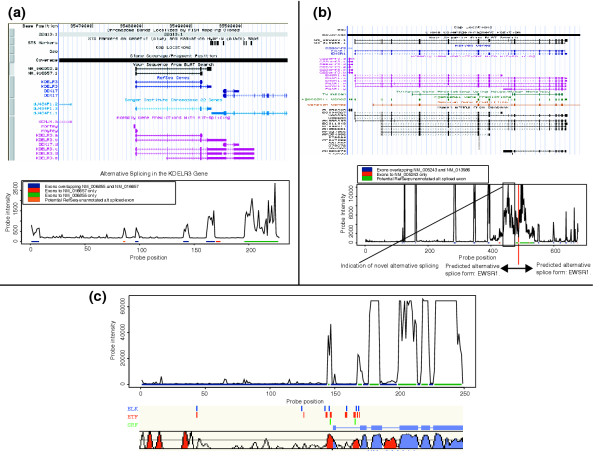
Examples of tiling results for known genes. The colored bars across the bottom of the data window are color matched with the corresponding exon annotations shown in the genome viewer. **(a) **The *KDELR3 *gene shows strong agreement between the public transcript annotations and the tiling results. The top panel represents a screen shot from the UCSC genome browser [60] highlighting *KDLER3*. The bottom panel represents transcription activity as raw intensities (*y*-axis) for each probe used to tile through *KDLER3 *(*x*-axis), in one of the eight conditions monitored by the genomic tiling arrays. **(b) **The *EWRS1 *gene potentially contains a larger number of false-positive predictions, but more probably lends additional experimental support to previously predicted alternative splice forms (*EWSR*.b and *EWSR*.g), giving a more accurate representation of the putative structure of this gene. The top panel represents a screen shot from the UCSC genome browser [60] highlighting EWRS1. The bottom panel represents transcription activity as raw intensities (y-axis) for each probe used to tile through EWSR1 (x-axis), in one of the eight conditions monitored by the genomic tiling arrays. **(c) **Conserved regions between mouse and human upstream of the beta-actin gene. The tiling data readily detect all of the transcribed parts of the gene, but not the conserved regulatory regions. The green bars in the probe-intensity plot represent the annotated transcribed regions for the beta-actin gene, while the blue bars indicate regions that are not known to be transcribed. The lower section shows the sequence conservation between human and mouse as obtained through the program rVISTA [36,61]. Conserved coding (blue peaks) and non-coding regions (red peaks) are shown where the two genomic sequences align with 75% identity over 100-bp windows. The rows marked ELK, ETF, and SRF show binding sites for these transcription factors predicted using TRANSFAC matrix models and the MATCHTM program, which are part of the rVISTA suite. The exons for the gene are shown in blue.

Figure 4 provides two representative examples of tiling data for two known Sanger genes, *KDELR3 *and *EWRS1*. In the first case (Figure 4a), the tiling data almost perfectly correspond to the RefSeq annotation of *KDELR3*, with just two potential false positives out of the 178 intron probes. The *KDELR3 *gene is annotated as having two alternative transcripts in the RefSeq database, given by the RefSeq accession numbers NM_006855 and NM_016657. The NCBI Acembly alternative splicing predictions further suggest the presence of additional isoforms of this gene (see Figure 4). One of the alternative forms, *KDELR3*.e, depicted in Figure 4a, includes a novel 5' exon. The presence of this exon is supported by the EST with GenBank accession number BM921831. The tiling data for the *KDELR3 *gene in two conditions clearly show expression of NM_006855 but not NM_016657, thereby reliably detecting distinct splice forms. Further, there is a significant signal 5' to exon 2 in both transcripts that seems to suggest a novel exon, as opposed to a true false positive. This putative exon exactly matches the location of the first exon given in the Acembly prediction track noted in Figure 4a (*KDELR3*.e).

Figure 4b shows the tiling data for the *EWSR1 *gene. In contrast to the first example, this gene has intense transcriptional activity outside of the annotated exons. Specifically, the *EWSR1 *gene has 43 potentially false-positive calls out of 203 intron probes. However, the EST data and alternative splicing predictions strongly suggest that these probes represent biologically relevant transcriptional activity. As with the *KDELR3 *gene, *EWRS1 *is annotated by RefSeq as having two transcripts: NM_005243 and NM_013986. The Acembly predictions identify four additional alternative splice forms; most noteworthy among these are *EWSR1*.b and *EWSR*.g, shown in Figure 4b. These predictions indicate that alternative transcripts may exist for the *EWSR1 *gene that essentially divide the largest transcript into two transcripts, suggesting that multiple promoter and transcription-stop signals are present in this gene. The tiling data depicted in Figure 4b shows that all exons from both RefSeq splice forms were detected. In addition, there is a region to the right of probe position 400 in Figure 4b that indicates significant transcription activity but where there are no RefSeq exons annotated. However, the green bars indicate exons that are supported by EST data as well as the *EWSR*.b and *EWSR*.g predicted alternative splice forms, providing experimental support that these predictions represent actual isoforms of this gene. In fact, these data may provide a more accurate representation of the putative structure of this gene, as they support multiple alternatively spliced transcripts in this gene, beyond what has already been annotated in the RefSeq database. In all, 5% of the probes detected as expressed in intronic sequence mapped to predicted alternative splice forms. Given the extent of alternative splicing that is yet to be characterized [21], we believe a significant proportion of the 'intron' transcriptional activity in our data may represent alternative splicing.

### Summarizing the tiling results

Our genome tiling arrays consisted of 2,119,794 and 1,201,632 probes for chromosomes 20 and 22, respectively. Of these, 1,615,034 probes fell into Sanger gene regions, with 239,542 probes actually overlapping Sanger exons. Under stringent criteria 64,241 probes were detected as expressed, with 34,245 of these falling within Sanger exons, 18,551 falling within Sanger introns, and 15,835 probes falling completely outside all Sanger annotations. This widespread transcriptional activity outside annotated regions of the human genome is consistent with other reports from multiple species [10,12,15,16]. Overall, at least one exon in each of 876 Sanger genes was detected as expressed out of 1,703 total genes covered by probes (excluding annotated pseudogenes), leading to an overall gene detection rate of 52%. The bias of probes identified as exon probes that actually fall in exons is striking, given that exons comprise roughly 2% of the genomic sequence (the *p*-value for this enrichment using the Fisher exact test is less than 10^-15^). To estimate the upper bound of false-positive calls, we counted as false-positive events each probe identified as expressed by the detection process, but falling within an annotated intron of the RefSeq genes we detected as expressed. This resulted in an estimated false-positive rate of 1.3%.

As indicated in Figure 4, a percentage of these false-positive calls will be due to unannotated isoforms of genes. Others still will be due to cross-hybridization of the intron probes to genes in other parts of the genome. We consider cross-hybridization as made up of two components: specific cross-hybridization resulting from transcripts with similar, usually homologous, sequences; and nonspecific cross-hybridization resulting from the base composition of the probe sequence (J.C. and G.C., unpublished work). Of the intron probes detected as expressed, 23% had sequence similarities to known transcripts considered to render them susceptible to specific cross-hybridization, and 17% contained sequence features associated with nonspecific cross-hybridization. Accounting for probes that were positive for both specific and nonspecific cross-hybridization, we are left with 55% of the probes detected as expressed in the introns of Sanger genes that cannot easily be explained as alternative splicing or cross-hybridization. These data support recent observations that significant levels of transcription exist within the introns of known genes [15,16].

For those probes falling outside all Sanger genes, we again made use of our custom genome annotations to help interpret the extent of transcriptional activity in these regions. Table 3 summarizes the detections made for each of the categories described above. Filtering probes using the same cross-hybridization predictors described above suggests that 65% of those probes falling outside all annotations are not likely to be the result of cross-hybridization. Furthermore, for those detections that overlap low-confidence locus projections in our PTI, we used the classification procedure discussed above to assign GO codes to these transcripts. Only seven of the 297 transcribed regions detected outside of all Sanger genes via the tiling results (see Table 3) contained GO protein domains. This suggests that a large fraction of the transcriptional activity detected using tiling arrays is non-coding and of unknown biological function [15,34].

### Tiling data help classify conserved sequences between species

One further advantage of the tiling data is that they can be used to discriminate between transcribed and non-transcribed sequences conserved between human and mouse, or between any other pair of species. Figure 4c highlights tiling data under one condition for the beta-actin gene, a gene that is constitutively expressed in all tissues and often serves as a positive control in mRNA and protein expression experiments. The genomic region containing the complete beta-actin mRNA and 10 kilobases (kb) of genomic sequence upstream of the transcription start, was obtained from the mouse and human genomes, aligned using the AVID program [35] and then fed into the rVista program [36]. From this, we identified the conserved regions in this gene between mouse and human, including several relevant transcription factor binding domains that are key to the transcriptional regulation of this gene [37-39]. As can be seen directly from the figure, the exons are all detected as highly expressed, but none of the conserved transcription factor regions shows activity. This combination of expressed sequence in close proximity to conserved regions that are not expressed (as determined by the tiling data), offers another powerful advantage of the tiling data in discriminating among the possible roles of conserved sequences.

## Discussion

A complete understanding of the human genome will only come after all genes have been identified and the functions of those genes have been determined. There has been much recent progress in defining the human transcriptome with *ab initio *methods, sequencing of EST libraries, full-length gene cloning projects, and comparative analyses between fully sequenced genomes of different species. However, we are still a long way from having a comprehensive set of annotations for the human and other genomes. There is need for new high-throughput experimental approaches to accelerate the process of annotating sequenced genomes in a comprehensive and accurate fashion. Toward this goal, we have used two microarray-based experimental approaches to provide evidence of widespread transcription activity outside of any known or predicted genes in the human genome. We have also provided experimental support for many *ab initio *predicted genes that have no other or minimal experimental sequence support, suggesting a small but significant class of genes that have evaded all other forms of experimental detection. Similar identifications have been made recently in the first extensive comparative analysis between mouse and human genomes [18]. Despite the extent of novel discovery, our data suggest there are only 25,000-30,000 protein-coding genes in the human genome, with perhaps an equal number of non-coding transcripts that may serve important regulatory roles [34,40]. Finally, our data indicate widespread alternative splicing across known genes, providing a glimpse into the extent of transcript complexity that may exist in mammalian genomes.

We have used the expression data for the approximate 50,000 predicted transcripts hybridized to 60 diverse conditions in combination with genomic tiling data to generate a CTI containing 28,456 experimentally supported transcripts. The transcripts represented in the CTI include all computational predictions with two or more lines of evidence from our PTI (independent of microarray validation), in addition to the overlapping set of 15,642 transcripts detected as EVGs. This resulting comprehensive list of known and predicted transcripts provides the starting point for large-scale systematic studies to determine the biological function of genes in both normal and disease states. The primary goal of the CTI is to allow researchers to focus experimental efforts on a comprehensive set of genes that are likely to be real.

It is of note that between the time the predicted transcript arrays were designed and annotated using the custom genome annotations described above, and the time this work was published, more than 6,000 genes were added to the RefSeq collection. These newer RefSeq genes were represented by 5,100 locus projections in our original PTI that were not classified in the RefSeq category. Interestingly, 4,212 were detected as EVGs in the present analysis and had already been included in our CTI, a validation rate slightly greater than 82%. Only 19% of the non-RefSeq genes in our PTI had been detected as EVGs (see Table 2), yet more than 82% of the new RefSeq genes coming from this set were detected as EVGs. This result speaks to the utility of the microarray-based approach to gene validation described here (see Additional data file 5 for a complete breakdown of validation rates by category).

While using microarrays to test computational gene predictions experimentally has the advantage of being economically feasible across the whole genome, the tiling data represent a more comprehensive and unbiased view of transcription. Our data indicate widespread transcriptional activity in the introns of annotated genes and in intergenic regions, where a significant proportion of this activity can be explained by nonspecific and specific cross-hybridization. The transcriptional activity noted for our low-onfidence transcripts in the PTI indicates that 25% of the activity we observe may be coding for proteins that are at least somewhat related to existing protein data. Much of the transcription activity we have noted in the introns of genes may also represent uncharacterized alternative splicing, and potentially novel genes, in addition to specific and nonspecific cross-hybridization.

## Conclusions

At present, predicted transcript arrays allow for the discovery of most protein-coding genes genome wide when many different conditions are considered. Until the discovery and characterization of these protein-coding genes is completed, this method will continue to be a cost-effective solution to drive such discovery. In contrast, genomic tiling represents a completely unbiased method for monitoring transcriptional activity in genomes, but due to cost will probably be limited to screening a smaller number of conditions. However, as novel transcription regions are identified from the tiling data, these regions can be represented on predicted transcript arrays that are hybridized over many more conditions, as described in Figure 1. As the microarray technologies have evolved, tiling the entire human genome is now possible, with such efforts presently being supported by the ENCODE (Encyclopedia of DNA Elements) project of the National Human Genome Research Institute (NHGRI) [41].

We believe the steps taken here are necessary for querying all potential transcription activity in the genome, for the purpose of identifying novel genes, more completely characterizing existing genes, and identifying a more comprehensive set of probes for these genes that can be used to monitor transcription abundances in more standard gene expression studies. Not all uses of microarrays demand an exhaustive representation of probes to all genes in the genome under study. However, experiments that seek to identify key drivers of pathways [42] or that seek to discriminate between alternative splice forms of genes within a given tissue [21] require a more comprehensive set of arrays to ensure success. These data provide an essential first step to generating a comprehensive set of arrays that are based on experimental support combined with computational annotation, instead of relying solely on the latter. These comprehensive arrays will be invaluable as we seek to better understand mechanisms of action for existing and novel drug targets and elucidate pathways underlying complex diseases. In addition, further study of the extensive noncoding RNA identified via the methods described here and elsewhere [10,12,15,16] is likely to open new fields of biology as the functional roles for these entities are determined.

## Materials and methods

### Data preparation

The NCBI 8/2001 assembly of the human genome was the input data for this analysis. The 4/21/1999 release of RepeatMasker [43] was used to mask for human repeats. An internal database of RNA genes and bacterial and vector sequences was aligned to the genome with BLASTN. Genomic sequences with 95% or higher identity over at least 50 bases were masked. No probes were designed from masked regions.

### Gene index production

To predict genes on the basis of expressed sequence similarity, we first clustered and aligned all expressed human and mouse sequences in GenBank to create a human gene index (HGI) and a mouse gene index (MGI). Clustering and alignment were performed with the DoubleTwist Clustering and Alignment Tools (CAT) [44]. Input data included all mouse and human RefSeq mRNA sequences, and EST and mRNA sequences from GenBank release 124, masked as described above for repeats and contaminating sequences. For each species, the CAT software first clustered sequences and then defined subclusters on the basis of a multiple sequence alignment. The subclusters represent candidate alternatively spliced gene transcripts. The 644,168 human and 291,656 mouse sequences that were singleton ESTs or completely masked were excluded from the HGI and MGI.

### Expressed sequence mapping

Human and mouse UniGene and RefSeq, MGI, and HGI sequences were aligned with the genome first by BLASTN 2.2.1 [45], followed by refinement of intron/exon boundaries by the sim4 algorithm (12/17/2000 release) [46]. Only the representative sequences (Hs.seq.uniq) for each UniGene cluster designated in the 3 August 2001/build 138 version of the UniGene database were used in this analysis. We only refined BLAST alignments with an E-value of less than 10^-20 ^for human sequences and 10^-8 ^for mouse sequences. For human UniGene and HGI, we refined only those BLAST hits where the target sequence showed greater than or equal to 92% identity to the genomic sequence over 75 bp. For human RefSeq, we refined hits with greater than or equal to 95% identity, and for MGI, RefSeq, and UniGene, we refined hits with greater than or equal to 80% identity. These thresholds were empirically determined to provide good sensitivity in aligning most sequences to the genome while limiting multiple alignments past those expected from paralogs present in the human genome. In all cases percent identity was measured over 75 bp. Individual sim4 exons of questionable confidence were then removed on the basis of percent identity and length thresholds. All sequence databases were downloaded from GenBank August, 2001.

### Protein sequence mapping

The GenBank nonredundant protein database (downloaded 25 August 2001) was aligned to the genomic sequence with BLASTX 2.2.1 [45] using an E-score threshold of 10^-5^. Adjacent protein alignments from a single protein were grouped together as a prediction whenever the protein sequence coordinates of the alignments were consistent in direction and did not significantly overlap.

### *Ab initio* gene prediction

GrailEXP 4.0 [47], GENSCAN 1.0 [48], FGENESH [49], and FGENESH+ [49]*ab initio *gene-prediction algorithms were run independently across the entire genome assembly to augment alignment-based gene identification methods. GrailEXP 4.0, GENSCAN 1.0, and FGENESH version 1.c were run with default parameters for human sequence. GrailEXP used expressed sequence evidence from RefSeq, UniGene and DoubleTwist HGI to refine gene predictions. FGENESH+ was run with protein sequences from BLASTX with E-score lower than 10^-5^. When multiple protein alignments overlapped, all overlapping protein sequences were clustered with BLASTClust [50] and the lowest E-score hit was used by FGENESH+.

### Synthesis and analysis

Locus projections contained the union of all exons from all overlapping predictions in a contiguous region of the chromosome that were derived from sequence alignments or gene-finding algorithms. Predictions to a given strand of the genomic sequence that overlapped by even a single nucleotide were grouped into a single locus projection (antisense transcripts were not considered in defining the locus projections). The criteria for grouping predictions were intentionally kept loose, given that the intent was to include as many potential exons as possible in a given genomic region, and then use the experimental microarray-based approach to elucidate the actual gene structure. These merged overlapping predictions defined the 5' and 3' ends of the locus projections. Overlapping predicted exons were merged to form an exon prediction of maximal extent. Low-quality predicted exons from sim4 alignments that contained a high percentage of A or T were removed. We also removed sim4-predicted exons that overlapped two or more predicted exons from another sim4 alignment. Additionally, 3' sim4 and 3' or 5' FGENESH+ predicted exons that were short and/or distant from internal predicted exons were removed. Finally, locus projections that contained mRNAs from RefSeq were split at the 5' end of the RefSeq sequence.

Locus projections supported by expressed sequences alone could be portions of 3' or 5' UTRs of genes included in the other gene-prediction categories described in the text. To minimize the consequences of this potential artifact, we used a UTR filter to exclude locus projections from the expressed sequence alone category that were within 20 kb of a locus projection supported by an *ab initio *gene model.

All data were loaded into a relational database to count and categorize locus projections. At least one type of evidence was assigned to each predicted exon for each locus projection. Multiple types of evidence were assigned to a merged predicted exon if there was overlap between predicted exons of different types for at least 1% of the length of the merged exon prediction. One of the eight evidence categories discussed in the text was assigned to each exon on the basis of the combination of types of evidence. Locus projections inherited the highest-ranking evidence category of their constituent exons. Evidence categories were ranked in the following order: Refseq (highest); expressed sequence + protein + *ab initio*; expressed sequence + *ab initio*; protein + *ab initio*; expressed sequence + protein; *ab initio *alone; protein alone; expressed sequence alone. FGENESH+ predictions were counted as protein + *ab initio*. For the *ab initio *category, predictions from at least two of FGENESH, GENSCAN and GrailEXP were required to overlap in at least one exon to be merged.

### Probe selection for the genome tiling and predicted transcript arrays

Input sequences for probe selection were masked for vector, interspersed repeats, simple repeats, poly(A) tails, *Escherichia coli *contamination and human non-coding RNA and mitochrondrial DNA contamination using Scylla (Paracel). For genomic tiling arrays, 60 mer probes were then selected from unmasked regions of both forward and reverse complement strands at uniform 30-base intervals. For predicted transcript arrays, up to four oligonucleotide probes were selected from the unmasked regions of each transcript using a multistep process.

The first step in the probe-selection process was the generation of a pool of candidate probes 60 nucleotides long (60 mers), where each probe was required to fall entirely within an exon from the set of exons under consideration. If there were fewer than four 60 mers then all 50 mers were considered as well. If there were fewer than four 50 mers or 60 mers then all 40 mers were considered, and so on. Stilts composed of sequence from *Saccharomyces cerevisiae *were added to the 3' ends of probes shorter than 60 nucleotides so that they had a total length of 60 bases when printed onto the arrays.

The second step in the probe-selection process was the classification and reduction of the probe pool on the basis of base composition and related filters. Probes were sorted into four classes on the basis of several criteria, including A, G, C and T content, GC content, the length of the longest homopolymeric run and the number of A residues at the 5' end. For example, a probe had to have GC content between 35 and 45% to be in class 1, between 15 and 55% to be in class 2, and between 10 and 60% to be in class 3. After all classifications were made, probes from lower-quality classes were discarded, keeping the number of probes per gene greater than 15. In cases where a pair of probes was overlapping by more than 50 bases, only a single probe was chosen.

The final step in the probe-selection process identified probes with minimal overlap, and predicted cross-hybridization and desirable positions in the transcript sequence. Cross-hybridization prediction was based on BLAST searching of the full collection of transcript sequences [51]. Probes with perfect matches to transcript sequences for genes other than the one undergoing design were discarded unless they were the only probes available. Otherwise the probes with the weakest predicted cross-hybridization interactions were preferred. Probes were also selected to have as little overlap as possible, and probes located in the last 500 bp of each transcript were discarded where possible to reduce the effects of impaired amplification in this region [52].

All arrays included a set of standard control probes which were used for image processing and quality control. Each array also included 30 randomly distributed copies of each of 51 negative-control probes. These probes were selected for their low intensities in previous human hybridizations. The negative controls local to each experimental probe were used for background correction. Non-control probes were added to each array such that all probes for a given input sequence were grouped together and ordered by their position on the sequence.

### Preparation of labeled cDNA and array hybridization

Hybridization material was generated through a random-priming amplification procedure using primers with a random sequence at the 3' end and fixed motif at the 5' end. This amplification procedure has been fully described [52] and has been optimized to generate strand-specific cDNA copies of the mRNA transcripts that are full-length. The 60 mRNA samples from the human tissues described in Additional data files 2 and 3 were purchased from Clontech. The 60 mRNA samples hybridized to the predicted transcript set of arrays were done in duplicate with fluor reversal to systematically correct for dye bias. For tiling hybridizations, six samples were used for chromosome 20 arrays and eight samples for chromosome 22. The mRNA samples hybridized to the set of tiling arrays were not done in duplicate as the analysis carried out on these data was intensity based, and our preliminary data demonstrated reasonable results without performing the tiling experiments in fluor-reverse pairs (data not shown). Additional data files 2-4 contain the full list of samples used for each set of arrays.

Array images were processed as described [53] to obtain background noise, single channel intensity and associated measurement error estimates. Expression changes between two samples were quantified as log_10 _(expression ratio) where the expression ratio was taken to be the ratio between normalized, background-corrected intensity values for the two channels (red and green) for each spot on the predicted transcript arrays. An independent normalization routine was carried out on the tiling data as described [33] to correct for dye biases, given the lack of technical replicates for these data.

### Analysis of predicted transcript array data

Probes from each computationally determined locus were analyzed for coordinated expression over 60 tissues by adapting an additive, probe-specific model initially developed to estimate gene expression indices [27]. The model for a single probe in a single sample pair is given by

*y*_*ij *_= *μ *+ *φ*_*j *_+ *θ*_*i *_+ *ε*_*j*_,

where the *y*_*ij *_represent the mlratio measurements for sample pair *i *and probe *j *in the current transcriptional model, *μ *is the grand mean term, *φ*_*i *_is the probe-specific term for probe *j *in the model, *θ*_*i *_is the sample-specific term for sample *i*, and *ε*_*j *_is the probe-specific error term, which is taken to be normally distributed with mean 0 and variance . Given the above representation for an observed mlratio value, the likelihood for a single probe over *N *condition pairs is simply



From this, the likelihood for a given transcriptional model, where a transcriptional model in this context is defined as a set of probes that are adjacent to one another in the genomic sequence and that co-regulate over a number of conditions, is easily seen to be the product of the individual probe likelihoods defined above over the *M *probes comprising the current model:



The maximum likelihood estimates for the parameters of this model are obtained using standard optimization techniques.

With the likelihood model described above, probe groups making up a transcriptional model were formed by iteratively considering whether neighboring probes (within a PTI member based on genomic location) of a given probe improved the fit of the model just described. This was determined by examining the likelihood ratio statistics between the current, best transcriptional model with or without an additional probe included in the model. Thresholds for the likelihood ratio test statistic and the different model parameters were empirically determined to minimize false-positive and false-negative rates. False positives were estimated by the detection of PTI members supported by only a single *ab initio *prediction that fell outside annotated Sanger genes on chromosomes 20 and 22. False negatives were defined as Sanger genes on chromosome 20 and 22 that were not detected. Probe sets with a maximum likelihood statistic greater than 100 and an *r*^2 ^value for fit of data to the model greater than 0.8 were considered high-confidence candidates for EVGs.

For each high-confidence EVG candidate, probes were further assessed by considering the number of conditions in which the absolute intensity of the probe was seen to be significantly above background, and the number of times the probe was seen significantly differentially expressed. Candidate EVGs with at least one probe that was: significantly above background (*p*-value < 0.01) in at least 10% of the samples; or significantly differentially expressed (*p*-value < 0.01) in at least 10% of the condition pairs, were considered validated.

### Analysis of tiling array data

The analysis of the tiling data has been described in detail by Ying *et al. *[33]. Briefly, probes were separated into 15 kb windows along the genome with 7.5 kb overlap between the windows. For each window, a robust principal component analysis was applied to the between-sample correlation matrix for probes in the window. Windows containing transcriptional activity were characterized by comparing the distribution of the Mahalanobis distances for the probes in the window (the Mahalanobis distance for each probe was calculated from the probe location to the center of the data in the first dimensions of the principal component score (PCS)) space with the reference distribution calculated based on known intron probes. Individual probes were then classified as belonging to the transcribed unit or not on the basis of the log of the Mahalanobis distance and an approximation of the diagonal distance (slope) of the probe from the minimum first PCS and median second PCS. Using these measures for distance, the probes were clustered using standard clustering techniques as described [33].

The procedure for estimating cross-hybridization of the probes is the subject of a separate manuscript. For the analyses described in this paper, the nonspecific cross-hybridization was estimated by the presence of motifs within the probe sequence that were enriched in probes observed to have a high level of nonspecific cross-hybridization. These probes were observed to have significant intensity when hybridized to human mRNA samples despite having no EST support and falling in introns of well characterized genes on chromosomes 20 and 22. Specific cross-hybridization was estimated by the minimum predicted ΔG value for hybridization of the probe to all genes other than the intended target in the UniGene database (build 157).

### Annotation of EVG and tiling data

Hidden Markov model Pfam (HMMPfam) domain predictions were run on six-frame translations of the PTIs using the HFRAME software from Paracel with an E-value cutoff of 0.01 and frameshift penalty of -12. Information on Pfam [31] domains is available [54,55]. GO terms [32] were then assigned to each locus projection using the full set of Pfam to GO mappings available from EBI FTP site [56]. The domain-to-ontology mapping is a part of the larger InterPro effort on annotating the proteome [57,58]. Multiple GO categories can be assigned to a single element of the PTI.

## Additional data files

The following additional data is available with the online version of this paper and at [19]. Additional data file 1 gives a complete list of 48,614 transcripts in the PTI that were represented on the set of predicted transcript arrays. Additional data file 2 gives a complete list of 60 tissues and cell lines hybridized to the predicted transcript arrays. Additional data file 3 gives a list of six tissues and cell lines hybridized to the chromosome 20 genomic tiling arrays. Additional data file 4 lists the eight tissues and cell lines hybridized to the chromosome 22 genomic tiling arrays. Additional data file 5  contains a comparison of EVG predictions with RefSeq sequences (March 2004). Also available on our website [19] are: ratio data and body atlas data along with the EVG status, and full sequences for the locus projections in fasta format. All probe sequences and expression data are available from the GEO database [59]. The series accession numbers for the tiling and predicted transcript arrays are GSE1097 and GSE918 respectively.

## Supplementary Material

Additional data file 1 A complete list of 48,614 transcripts in the PTI that were represented on the set of predicted transcript arrays Click here for additional data file

Additional data file 2 A complete list of 60 tissues and cell lines hybridized to the predicted transcript arrays Click here for additional data file

Additional data file 3 A list of six tissues and cell lines hybridized to the chromosome 20 genomic tiling arrays Click here for additional data file

Additional data file 4 The eight tissues and cell lines hybridized to the chromosome 22 genomic tiling arrays Click here for additional data file

Additional data file 5 A comparison of EVG predictions with RefSeq sequencesP Click here for additional data file

## Figures and Tables

**Table 1:** Comparison of locus projections in the PTI on chromosomes 20 and 22 to Sanger-annotated genes

		Sanger chromosome 20, genes	Non-Sanger chromosome 20, genes	Sanger chromosome 22, genes	Non-Sanger chromosome 22, genes
Sanger genes (including pseudogenes)		1,297		936	
					
Locus projection categories					
High-confidence categories	RefSeq	676 (30)	8	375 (47)	12
	*Ab initio *+ expressed sequence + protein	336 (63)	10	285 (127)	10
	*Ab initio *+ expressed sequence	38 (2)	96	28 (7)	74
	*Ab initio *+ protein	28 (11)	37	31 (18)	29
	Expressed sequence + protein	38 (30)	37	36 (30)	24
					
Low-confidence categories	*Ab initio*	22 (4)	674	50 (21)	362
	Protein	17 (14)	157	18 (13)	121
	Expressed sequence	22 (2)	1,591	31 (7)	1,127
					
Higher-confidence categories		1,116 (136)	188	755 (229)	149
All categories		1,177 (156)	2,610	854 (270)	1,759

Columns 1 and 3 provide the number of locus projections in the PTI set that overlap Sanger genes for chromosomes 20 and 22, respectively. The numbers given in parentheses indicate the number of Sanger-annotated pseudogenes; these pseudogenes were not used when summarizing the results. Columns 2 and 4 give the number of genes in the PTI set that were not overlapping Sanger genes.

**Table 2:** Summary of expression-validated genes (EVGs) from predicted transcripts over the entire human genome

Gene categories	Sanger/PTI chromosome 20	Non-Sanger PTI chromosome 20	Sanger/PTI chromosome 22	Non-Sanger PTI chromosome 22	PTI genome-wide
Total Sanger genes represented	1,177 (826)		854 (575)		
					
RefSeq	676 (552)	8 (2)	375 (290)	12 (5)	10,720 (7992)
*Ab initio *+ expressed sequence + protein	336 (229)	10 (2)	285 (202)	10 (5)	8,801 (4269)
*Ab initio *+ expressed sequence	38 (17)	96 (8)	28 (15)	74 (8)	3,733 (784)
*Ab initio *+ protein	28 (9)	37 (7)	31 (16)	29 (4)	1,983 (233)
Expressed sequence + protein	38 (2)	37 (2)	36 (10)	24 (4)	1,126 (271)
Expressed sequence	22 (3)	1,591 (44)	31 (3)	1,127 (33)	7,170 (1428)
*Ab initio*	22 (12)	674 (39)	50 (35)	362 (17)	16,822 (555)
Protein	17 (2)	157 (7)	18 (4)	121 (4)	540 (110)
					
High-confidence categories	1,116 (809)	188 (21)	755 (533)	149 (26)	26,363 (13,549)
All categories	1,177 (826)	2,610 (111)	854 (575)	1,759 (80)	50,895 (15,642)

Columns 1 and 3 provide the total number of Sanger genes for each category for chromosomes 20 and 22, respectively, with the number of EVGs detected given in parentheses. Columns 2 and 4 provide the total number of LPs that did not overlap Sanger genes, with the number of EVGs detected given in parentheses. The last column provides the total number of LPs in the PTI represented on the PTA microarrays, with the number of EVGs detected over the entire genome given in parentheses.

**Table 3:** Summary of transcription activity detected from the chromosome 20 and 22 genome tiling data

Locus projection categories	Sanger tiling chromosome 20	Non-Sanger tiling chromosome 20	Sanger tiling chromosome 22	Non-Sanger tiling chromosome 22
Total Sanger genes	1,278		933	
					
Sanger category 1	577 (398)		368 (184)	
Sanger category 2	155 (32)		121 (60)	
Sanger category 3	338 (150)		144 (52)	
Sanger category 4	161 (117)		294 (138)	
RefSeq		3		0
*Ab initio* + expressed sequence + protein		1		0
*Ab initio* + expressed sequence		15		8
*Ab initio* + protein		6		4
Expressed sequence + protein		4		1
*Ab initio*		71		26
protein		11		21
Expressed sequence		80		46
Outside all annotations*		1,936		1,058
High-confidence categories	NA	25	NA	12
All annotation categories	1,231 (697)	191	927 (434)	106

*Number of probes detected as components of EVGs. Columns 1 and 3 provide the number of Sanger genes represented on the genome tiling arrays for chromosomes 20 and 22, respectively, with the number of genes detected given in parentheses. Columns 2 and 4 provide the number of LPs not overlapping Sanger genes that were detected on chromosomes 20 and 22, respectively. NA, not applicable.
