# Supplementary material for: A comprehensive transcript index of the human genome generated using microarrays and computational approaches
Source: Genome Biol. 2004 Sep 23;5(10):R73. doi: 10.1186/gb-2004-5-10-r73 (PMC545593; doi:10.1186/gb-2004-5-10-r73)
Supplement: Additional data file 2 — A complete list of 60 tissues and cell lines hybridized to the predicted transcript arrays [file gb-2004-5-10-r73-s2.doc]

**Table S2.** Complete list of 60 tissues and cell lines hybridized to the predicted transcript arrays described in the main text.

|  | Organism | Sample Type | Sample Description |
| --- | --- | --- | --- |
| 1 | Human | Tissue | Adrenal Cortex |
| 2 | Human | Tissue | Adrenal Medulla |
| 3 | Human | Tissue | Bladder |
| 4 | Human | Tissue | Fetal Liver |
| 5 | Human | Tissue | Kidney |
| 6 | Human | Tissue | Prostate |
| 7 | Human | Tissue | Skeletal Muscle |
| 8 | Human | Tissue | Adrenal Gland |
| 9 | Human | Tissue | Bone Marrow |
| 10 | Human | Tissue | Brain Amygdala |
| 11 | Human | Tissue | Brain Caudate Nucleus |
| 12 | Human | Tissue | Brain Cerebellum |
| 13 | Human | Tissue | Brain Corpus Callosum |
| 14 | Human | Tissue | Brain |
| 15 | Human | Tissue | Brain Thalamus |
| 16 | Human | Tissue | Brain Cerebral Cortex |
| 17 | Human | Tissue | Brain Hippocampus |
| 18 | Human | Tissue | Brain Postcentral Gyrus |
| 19 | Human | Cell Line | Colorectal Adenocarcinoma (SW480) |
| 20 | Human | Tissue | Descending Colon |
| 21 | Human | Tissue | Duodenum |
| 22 | Human | Tissue | Epididymus |
| 23 | Human | Tissue | Fetal Brain |
| 24 | Human | Tissue | Fetal Kidney |
| 25 | Human | Tissue | Fetal Lung |
| 26 | Human | Tissue | Heart |
| 27 | Human | Tissue | Hela |
| 28 | Human | Tissue | Ileocecum |
| 29 | Human | Tissue | Ileum |
| 30 | Human | Tissue | Interventricular Septum |
| 31 | Human | Tissue | Jejunum |
| 32 | Human | Cell Line | Leukemia Chronic Myelogenous (K562) |
| 33 | Human | Cell Line | Leukemia Lymphoblastic (MOLT-4) |
| 34 | Human | Cell Line | Leukemia Promyelocytic (HL-60) |
| 35 | Human | Tissue | Liver |
| 36 | Human | Tissue | Liver Left Lobe |
| 37 | Human | Cell Line | Lung Carcinoma (A549) |
| 38 | Human | Tissue | Lung |
| 39 | Human | Tissue | Lymph Node |
| 40 | Human | Cell Line | Lymphoma Burkitt's (Daudi) |
| 41 | Human | Cell Line | Lymphoma Burkitt's (Raji) |
| 42 | Human | Cell Line | Melanoma (G361) |
| 43 | Human | Tissue | Pancreas |
| 44 | Human | Tissue | Placenta |
| 45 | Human | Tissue | Rectum |
| 46 | Human | Tissue | Retina |
| 47 | Human | Tissue | Salivary Gland |
| 48 | Human | Tissue | Small Intestine |
| 49 | Human | Tissue | Spinal Cord |
| 50 | Human | Tissue | Spleen |
| 51 | Human | Tissue | Stomach |
| 52 | Human | Tissue | Testis |
| 53 | Human | Tissue | Thyroid |
| 54 | Human | Tissue | Tongue |
| 55 | Human | Tissue | Tonsil |
| 56 | Human | Tissue | Trachea |
| 57 | Human | Tissue | Transverse Colon |
| 58 | Human | Tissue | Uterus |
| 59 | Human | Tissue | Uterus Corpus |
| 60 | Human | Tissue | Thymus |
