# Supplementary material for: A comprehensive transcript index of the human genome generated using microarrays and computational approaches
Source: Genome Biol. 2004 Sep 23;5(10):R73. doi: 10.1186/gb-2004-5-10-r73 (PMC545593; doi:10.1186/gb-2004-5-10-r73)
Supplement: Additional data file 4 — The eight tissues and cell lines hybridized to the chromosome 22 genomic tiling arrays [file gb-2004-5-10-r73-s4.doc]

**Table S4.** List of 8 tissues and cell lines hybridized to the chromosome 22 genomic tiling arrays described in the main text.

|  | Organism | Sample Type | Sample Description |
| --- | --- | --- | --- |
| 1 | Human | Tissue | Brain Thalamus |
| 2 | Human | Tissue | Fetal Kidney |
| 3 | Human | Cell Line | Jurkat |
| 4 | Human | Cell Line | Leukemia Chronic Myelogenous (K562) |
| 5 | Human | Tissue | Spleen |
| 6 | Human | Tissue | Testes |
| 7 | Human | Tissue | Thymus |
| 8 | Human | Tissue | Uterus |
