# Supplementary material for: A comprehensive transcript index of the human genome generated using microarrays and computational approaches
Source: Genome Biol. 2004 Sep 23;5(10):R73. doi: 10.1186/gb-2004-5-10-r73 (PMC545593; doi:10.1186/gb-2004-5-10-r73)
Supplement: Additional data file 5 — A comparison of EVG predictions with RefSeq sequencesP [file gb-2004-5-10-r73-s5.doc]

**Table S5.** Comparison of Expression Validated Gene (EVG) predictions with RefSeq sequences (March, 2004). The first column represents the predicted gene categories as described in the main text. The second column gives the counts of PTI genes mapping to the current RefSeq set by category that were detected as expression verified genes (EVG). The third column gives the counts of those PTI genes mapping to the current RefSeq set that were not detected as EVGs. The fourth and fifth columns are simply the percentages associated with the second and third column, respectively.

| Support Category | EVG | Non-EVG | EVG Percentage | Non-EVG Percentage |
| --- | --- | --- | --- | --- |
| Known (originally contained RefSeq gene) | 9672 | 1480 | 86.7 | 13.3 |
| cDNA, Protein, and Gene Model Supported | 2862 | 520 | 84.6 | 15.4 |
| Protein and EST Supported | 82 | 10 | 89.1 | 10.9 |
| cDNA Supported | 703 | 102 | 87.3 | 12.7 |
| Protein Supported | 39 | 4 | 90.7 | 9.3 |
| cDNA and Gene Model Supported | 319 | 102 | 75.8 | 24.2 |
| Protein and Gene Model Supported | 113 | 77 | 59.5 | 40.5 |
| Gene Model Supported by 2 Predictions | 48 | 53 | 47.5 | 52.5 |
| Gene Model Supported by 1 Prediction | 46 | 20 | 69.7 | 30.3 |
| Total | 13884 | 2368 | 85.4 | 14.6 |
| Total Excluding Known Category | 4212 | 888 | 82.6 | 17.4 |

**[Author: OK to include the following here?]** To further validate the expression verified genes identified in the analysis presented in the main text, all probes from the PTI were mapped to the most recent set of RefSeq genes. For a probe to be assigned to a RefSeq sequence, 56 out of 60 bases had to match the positive strand of the RefSeq sequence with no gaps. Probes with hits to multiple RefSeqs from different Locus Link records were discarded. All locus projections containing probes mapping to the current RefSeq set were then summarized based on the EVG detection status and original locus projection category presented in the main text. The results of this summary are given in Supplemental Table S5. Slightly more than 85% of the locus projections that mapped to the latest RefSeq gene set were detected as expression verified genes. This percentage is higher than expected based on the false negative predictions from the main manuscript, which suggests the estimates provided were somewhat conservative. However, because transcripts that are more highly expressed over a broader range of tissues are over represented in RefSeq and, therefore, are the easiest to detect using the microarray-based approach described in the main text, the conservative estimate provided in the main text is still warranted. We also note that there is an increased percent of EVGs in the known category (based on RefSeq alignments to the genome) for those locus projections matching current RefSeqs (87%), compared with the percentage of all locus projections referenced in the paper (75%). This is likely due to the removal of incorrect provisional RefSeq sequences during the review process and again highlights the value of microarray validation pending full characterization of the human transcriptome. The percentage of EVGs drops as the reliance on gene model predictions for the locus projection increases. This is likely due to cases where the structure of the gene model was incorrect. Since one of the major criteria for determining an EVG is co-regulation of probes across conditions, probes designed against incorrect portions of a partially correct gene model will reduce the power to detect that gene.
